# Juvenile Huntington’s Disease: A Case Report and a Review of Diagnostic Challenges

**DOI:** 10.7759/cureus.40637

**Published:** 2023-06-19

**Authors:** Su-Yuan Yu, Stormie Gough, Auguste Niyibizi, Muhammed Sheikh

**Affiliations:** 1 School of Medicine, University of South Florida Health, Tampa, USA; 2 Department of Pediatrics, Lehigh Valley Reilly Children’s Hospital, Allentown, USA; 3 Department of Family Medicine, St. Luke’s University Sacred Heart Hospital, Allentown, USA; 4 Department of Neurology, Lehigh Valley Reilly Children’s Hospital, Allentown, USA

**Keywords:** rare neurodegenerative disease, parkinsonism, developmental regression, juvenile huntington disease, medically refractory epilepsy, huntington disease

## Abstract

Juvenile Huntington’s Disease (JHD) is a rare variant of the hereditary neurodegenerative disorder Huntington’s disease (HD). Clinical symptoms in JHD are broad and non-specific, making the initial diagnosis difficult. In this report, we describe a young Hispanic male who gradually developed cognitive decline, dystonia, and seizures. His diagnosis was delayed despite multiple visits to his pediatrician, developmental specialist, and neurologist. A history of developmental regression and unusual imaging findings prompted genetic testing, which led to the diagnosis of JHD. Though changes in the striatum on MRI are hallmarks of JHD, family and developmental history often provide the most important diagnostic clues. Careful history-taking in patients with non-specific neurological exam findings, as in patients with JHD, can prevent diagnostic delays and allow for early interventions to improve quality of life.

## Introduction

Huntington’s disease (HD) is a rare hereditary neurodegenerative disorder characterized by chorea, cognitive decline, and psychiatric disturbances. The prevalence in the Caucasian population is estimated to be four to ten cases per 100,000 [1]. Most cases of HD manifest in adulthood between the third and fifth decade of life [2]. HD rarely occurs in children younger than 21 years of age, with an incidence of 0.7 per million patient years [1]. Unlike the adult-onset form, juvenile Huntington’s disease (JHD) typically presents with rigidity, bradykinesia, and seizures. Less specific features include learning difficulties, developmental regression, and behavioral problems [3,4]. Early signs of JHD are often overlooked due to the rarity of the disease and the nonspecific nature of the symptoms. There have been instances in which the behavioral problems caused by JHD were misdiagnosed as attention-deficit hyperactivity disorder (ADHD) and its involuntary movements as Tourette syndrome [4,5]. Studies have linked a younger age of onset to a longer diagnostic delay; JHD has a mean diagnostic delay of nine years [3].

The consequences of a delayed diagnosis include frequent hospital visits, inadequate treatment, and an added emotional burden on the family [6]. Although the diagnosis is terminal, supportive care can be provided to the patient to help optimize their quality of life. The care of a child with JHD often requires a multidisciplinary team of neurologists, psychiatrists, speech and physical therapists, dieticians, and special education teachers in school. In this report, we present a case of JHD with a delayed diagnosis secondary to multiple diagnostic challenges.

## Case presentation

An 11-year-old Hispanic male with a history of generalized epilepsy since age nine presented to the emergency department (ED) with breakthrough seizures. He had six episodes of generalized tonic-clonic seizures. Each episode lasted for three to five minutes. In the ED, he had another two episodes of witnessed tonic-colonic seizures that abated with lorazepam. The patient’s mother reported medication compliance and denied any preceding illnesses or head trauma. He was admitted to the pediatric intensive care unit a month prior for status epilepticus. Upon presentation, his vital signs were within normal limits. He demonstrated an abnormal neurological exam with dysarthria, cogwheel rigidity, hand tremor, and ataxia. An ophthalmologic exam revealed vertical gaze palsy and optic atrophy. The initial complete blood count and complete metabolic panel were unremarkable. Because of the abnormal neurological exam findings and breakthrough seizures, the patient was admitted for further evaluation and workup.

Careful developmental and family history was taken during this admission. It was discovered that the patient started walking at nine months of age. He spoke single words when he was 12 months old and started making two- and three-word phrases by the age of three. His developmental history was unremarkable until he was seven years old when he started to experience speech difficulties in school. His speech was intelligible to the immediate family 90 percent of the time but to extended family and friends only 30 percent of the time, raising concerns for developmental regression. A developmental psychologist evaluated the patient and expressed concerns about intellectual disability, articulation disorder, and ADHD. At eight years of age, the patient developed abnormal posturing in the left upper extremity with an asymmetric arm swing while walking and running. He subsequently developed seizures, for which he was diagnosed with epilepsy secondary to chronic static encephalopathy. Despite treatment with valproic acid and zonisamide, he continued to experience breakthrough seizures in the setting of progressive neurological decline.

The child’s family history was significant because of the premature death of the father. The mother states that the father was healthy when she met him at the age of 18. They separated shortly after the patient was born because of the father’s frequent involvement in physical fights outside of the home. Beginning in his twenties, the father suffered from gait instability and frequent falls, eventually becoming bedridden. He also developed intractable seizures. His neurological deterioration was attributed to repeated traumatic brain injuries. At the age of 30, he died of an intracranial hemorrhage after a fall.

**Figure 1:**
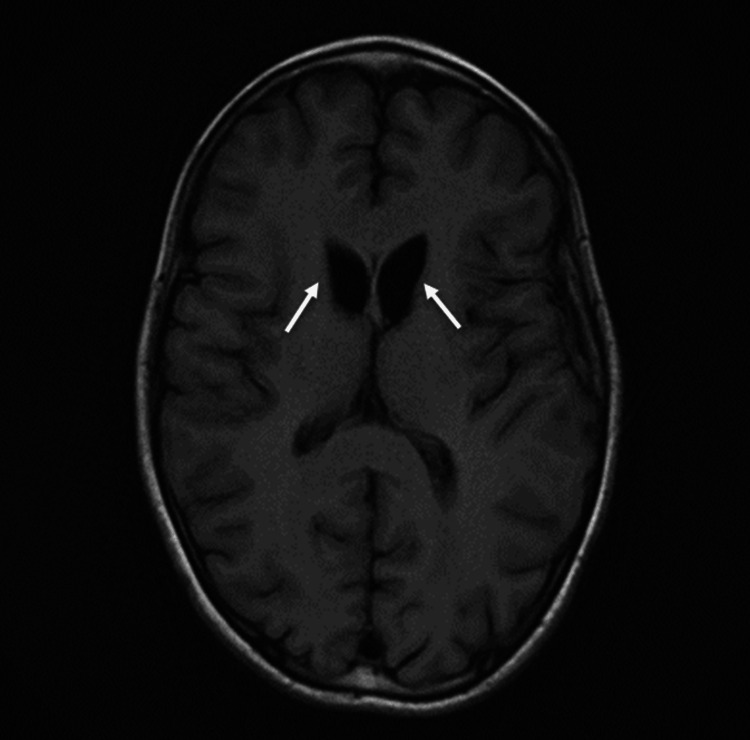
Axial T1-weighted MRI of the brain shows volume loss in the bilateral putamen and caudate heads (white arrows).

**Figure 2:**
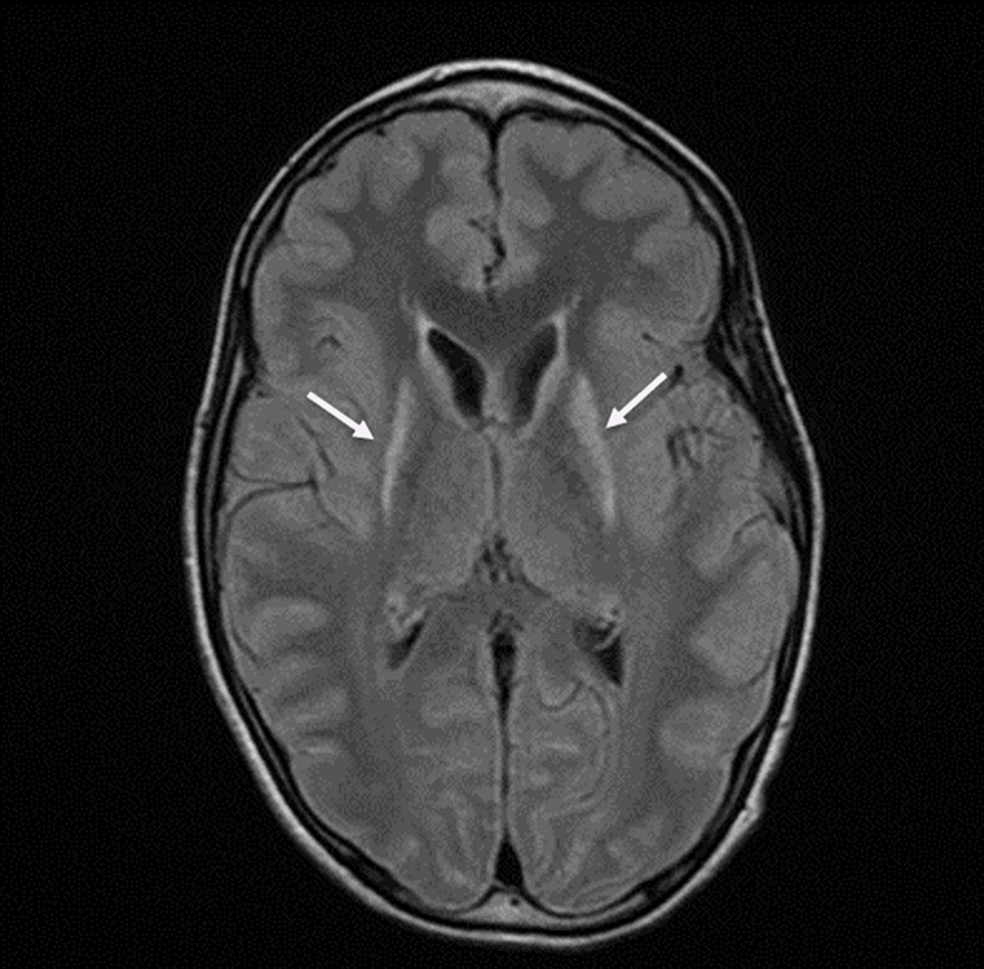
Axial FLAIR MRI of the brain shows symmetric areas of abnormal T2 hyperintensity involving the bilateral putamen (white arrows).

A prior MRI study done at age eight showed symmetrical signal abnormalities in his bilateral putamen, which were attributed to a remote perinatal injury. The patient’s recent continuous EEG showed nonspecific spike and wave patterns with no evidence of seizure activity. Because of the patient’s developmental regression and family history, a repeat MRI of the brain was performed. It showed significant volume loss in the bilateral caudate heads associated with symmetric T2 hyperintensity in the putamen (Figure 1, 2). This is suggestive of neurogenerative disorders.

Laboratory studies for serum lactate, ammonia, pyruvate, plasma amino acids, carnitine, acylcarnitine, organic acids, copper, mitochondrial DNA sequencing, and ceruloplasmin came back normal. Polymerase chain reaction (PCR) genetic testing for HD mutations revealed 92 CAG repeats in HD allele 1 and 17 repeats in HD allele 2. The diagnosis of JHD was disclosed to the family in the presence of a genetic counselor and social worker. A multidisciplinary team was created to care for the child.

## Discussion

HD is an autosomal-dominant hereditary disease caused by the expansion of CAG trinucleotide sequences within the HTT gene, which encodes for huntingtin protein (HTT) [7]. Although the disease mechanism is still not completely understood, it is thought that the mutant huntingtin protein (mtHTT) results in preferential degradation of striatal GABAergic medium-sized spiny neurons, leading to neuronal deaths within the striatum (the region responsible for the coordination of movement) [5,8]. The larger the CAG expansion, the younger the age of onset and the faster the rate of disease progression [7]. Patients with JHD typically have more than 60 repeats [9]. Our patient’s early onset of disease can be explained by the large number of CAG repeats in his HD allele 1.

Diagnosing JHD is difficult due to its rare occurrence and variable presentation. Its phenotypic range overlaps with many other childhood neurodevelopmental and neurodegenerative disorders, such as dentatorubral-pallidoluysian atrophy, juvenile-onset Parkinson’s disease, heavy metal toxicities, mitochondrial disorders, neuroacanthocytosis, pantothenate kinase-associated neurodegeneration, spinocerebellar ataxia, and Wilson’s disease [10,11]. Because phenotypic features can be broad and encompassing, a detailed history can offer important clues to the diagnosis. The patient’s developmental regression-the process of losing acquired milestones-is appropriately differentiated from developmental delay, defined as not achieving or advancing to new developmental milestones.

In addition to the developmental history, early paternal death was a red flag in our initial assessment of our patient. It has been found that a family history of HD is the most sensitive and specific predictor of genetic status [12]. The inheritance of the HTT gene is governed by anticipation, with trinucleotide repeats expanding with each successive generation. The age of onset of the offspring often occurs earlier than that of the affected parent, particularly when passed through the male germline [13]. The parents of our patient separated when the patient was young. Initially, the mother did not volunteer information regarding the biological father’s disease history. Only later investigation with the paternal grandmother gave more clarity to the father’s rapid neurological deterioration and premature death at the age of 30, strongly suggestive of a history of neurodegenerative disease.

Our patient was developmentally normal until the age of seven when he reportedly started to show speech difficulties, extremity posturing, irregular saccades, and a learning disability. His condition worsened precipitously within a few years, with the development of profound parkinsonism and intractable seizures. It is documented that symptoms such as cognitive dysfunction occur in 60-70 percent of cases; rigidity in 30-50 percent; and behavioral changes and difficulties in 87 percent. Chorea is rare, presenting in only 10 percent of JHD cases [14,15]. Seizures occur in 38 percent of individuals with JHD, which may arise from primary neuronal degradation in the striatal synapses [11].

Detailed history-taking not only helps on a clinical diagnostic level but also assists the radiologist in generating a robust list of differential diagnoses based on the imaging findings. Though image findings may appear late in the disease process, changes in the striatum-composed of caudate, putamen, and ventral striatum nuclei-are the hallmark of the disease process [13,14]. These classic MRI findings have been observed in our case, which show volume loss in the bilateral putamen and caudate heads and increased T2 intensities in the bilateral basal ganglia, which include the striatum.

The diagnosis of JHD is definitive, and currently, there are no curative or disease-modifying treatments [15]. The recommended approaches are behavioral interventions that target symptom control. These include an antidepressant for depression and anxiety, seizure medication for seizure activity and mood stabilization, carbidopa or levodopa for parkinsonian-like features, baclofen for rigidity, and alpha-agonists for impairment in executive function and impulsivity [15]. Tetrabenazine has been approved by the U.S. Food and Drug Administration for chorea. Antipsychotics such as risperidone are helpful for behavioral problems and neuropsychiatric manifestations. At late stages, patients may become bedridden and require around-the-clock nursing care [3]. Supportive care with physical, speech, and occupational therapy is essential for maximizing functionality [5].

## Conclusions

JHD is a devastating disease due to its rapidly progressive nature and the heavy care burden it places on the family. A delay in diagnosis, as found in our case, can be an additive source of stress for the patient and their family. A detailed family and developmental history provide the most important diagnostic clues for a patient with concerns about neurodegenerative diseases. A multidisciplinary approach should be adopted, as timely imaging and expert opinions from neuroradiologists can significantly improve diagnostic accuracy. Early diagnosis of JHD will allow the patient to receive appropriate care and achieve a better quality of life.
